# Gender Differences in Transdiagnostic Predictors of Problematic Alcohol Consumption in a Large Sample of College Students in Ecuador

**DOI:** 10.3389/fpsyg.2022.784896

**Published:** 2022-02-24

**Authors:** Rafael Sánchez-Puertas, Pablo Ruisoto, Carla López-Núñez, Silvia Vaca-Gallegos

**Affiliations:** ^1^Department of Health Sciences, Public University of Navarre, Pamplona, Spain; ^2^Department of Psychology, Universidad Técnica Particular de Loja, Loja, Ecuador; ^3^Department of Personality, Assessment and Psychological Treatments, School of Psychology, University of Seville, Seville, Spain

**Keywords:** alcohol, gender differences, transdiagnostic predictors, college students, psychological stress, psychological inflexibility, Ecuador

## Abstract

**Background:**

Alcohol use is one of the main risk factors that leads to detrimental health effects and support for a transdiagnostic approach to alcohol use disorders is growing. However, the role of transdiagnostic predictors of problematic alcohol consumption in Ecuador are understudied.

**Objective:**

The aim of this study was to examine gender differences in psychological stress and inflexibility as transdiagnostic predictors of problematic alcohol consumption in a large sample of college students in Ecuador.

**Methods:**

A total of 7,905 college students (21.49 years, SD = 3.68; 53.75% females) were surveyed using the following standardized scales: Alcohol Use Disorders Identification Test, Perceived Stress Scale-14, and Acceptance and Action Questionnaire (AAQ-7). Macro Process for SPSS (models 4 and 7) was used to analyze mediation and moderation effects.

**Results:**

Reported alcohol consumption was significantly higher in men than women students. On the other hand, women reported significantly higher levels of perceived stress and psychological inflexibility than men students. Gender, age, psychological stress, and inflexibility were significant predictors of alcohol consumption. Moreover, psychological inflexibility mediated the impact of stress on alcohol consumption, particularly in women (for men *b* = 0.065, 95% CI [0.048 to 0.083], for women *b* = 0.070, 95% CI [0.051 to 0.089]).

**Discussion:**

Results of this study support psychological stress and psychological inflexibility as critical transdiagnostic variables related to increased rates of alcohol consumption among Ecuadorian college students. These conclusions contribute to the development of transdiagnostic comprehensive programs, which encompasses promotive, preventive, and treatment services that allow to alleviate the burden of alcohol, as well as to enrich the growing research on alcohol consumption in this population from a gender perspective.

## Introduction

According to the Global Status Report on Alcohol and Health 2018, among young adults between the ages of 20–39 years old, approximately 13.5% of all deaths are the result of alcohol consumption; moreover, the peak of alcohol consumption in young adults falls between the 20 and 24 years (World Health Organization, 2018), corresponding with college ages. In Ecuador, based on the latest Report on Drug Use in the Americas, 2019, prevalence of alcohol consumption in the last month among college students is 51%, and a total of 41% of alcohol users reported problematic levels of consumption, systematically higher in male than female college students (Organization of the American States, 2019).

Despite the great influence of diagnostic approaches to alcohol use disorders (AUD) and other-related mental health problems, such as depression and anxiety (Castillo-Carniglia et al., 2019; McHugh and Weiss, 2019), some studies argue that psychiatric gnosologies must be updated to fulfill their purpose in research and clinical practice (Carragher et al., 2015; Hofmann and Hayes, 2019). Therefore, a new transdiagnostic approach is proposed, which goes beyond the traditional diagnostic limits, and can provide a new perspective into how to account for AUD and other mental health problems, thus having a better classification system, compared to the existing standard (Dalgleish et al., 2020). This new approach arises from a series of findings that suggest that the commonalities between the different disorders should be taken into account for evaluation and treatment (Nolen-Hoeksema and Watkins, 2011).

In this context, some transdiagnostic factors have drawn special attention in AUD and other mental health-related problems, being psychological (in)flexibility one of the key transdiagnostic clinical variables. This construct, also known as experiential avoidance, is defined as the tendency to control, avoid, or suppress the intensity and/or frequency of thoughts and feelings, even when doing so generates behavioral harm (Hayes et al., 1996), and may contribute to the development, maintenance, and exacerbation of AUD among college students (Levin et al., 2012). Second, psychological stress also plays a crucial role within this transdiagnostic emotional vulnerability model, and it is defined as a subjective perception of not being able to control or foresee the results of our behavior (Cohen et al., 1983), which has also been related to AUD in college students (Anthenelli, 2012; Keyes et al., 2012; Karyotaki et al., 2020).

Previous studies have warned about the particularly high and continuously growing rates of problematic alcohol consumption among college students in Ecuador (Ruisoto et al., 2016; Aguilar et al., 2022), but successful assessment models accounting for this problematic behavior and gender differences remain a challenge, in particular how key transdiagnostic variables moderate and/or meditate alcohol consumption (Nolen-Hoeksema, 2004; Dodd et al., 2010; Heidari et al., 2018). With the purpose of overcoming this gap in the literature, the aim of this study was to examine gender differences in psychological stress and inflexibility as transdiagnostic predictors of problematic alcohol consumption in a large sample of college students in Ecuador. Furthermore, we examined whether psychological inflexibility and psychological stress mediate the relationship between gender and alcohol consumption, and whether psychological inflexibility mediates the relationship between stress and alcohol consumption, moderated by gender.

## Materials and Methods

### Participants

Students enrolled at 11 Ecuadorian universities were invited to participate in the study by email, completing a computerized survey within a 3-week assessment period. A total of 7,905 participants (average age was 21.49 years, SD = 3.68; 53.75% females) met the inclusion criteria of being enrolled in a full academic year and having completed the entire survey. The average response rate across universities was 47.80%, ranging from 39.10 to 56.35%. All participants gave their written informed consent before their enrolment in the study. The majority of students was single (91.3%), they belonged to the Highlands region (63.49%), and their main occupation was being students (73.38%). For a detailed description of sociodemographic measures by gender, see Table 1.

**Table 1:** Gender differences in sociodemographic variables.

Variables	Men M (SD) (*n* = 3,656)	Women M (SD) (*n* = 4,249)	*t*	*p*	Cohen’s *d*
Age (years)	21.79 (3.68)	21.24 (3.68)	6.66	<0.001^***^	0.15
	**Men % (n = 3,656)**	**Women % (n = 4,249)**	**Chi Square**	***p***	**Cramer’s V**
Geographical region (A/C/H)	9.21 (337)/23.25 (850)/67.53 (2469)	9.86 (419)/30.15 (1281)/60.0 (2549)	53.155	<0.001^***^	0.082
Marital status (S/M/D/W)	43.18 (3413)/2.85 (225)/0.22 (17)/0.01 (1)	48.17 (3808)/5.1 (400)/0.051 (40)/0.01 (1)	35.62	<0.001^***^	0.067
Main occupation (St/SW)	32.52 (2571)/13.73 (1085)	40.86 (3230)/12.9 (1019)	32.63	<0.001^***^	0.064

M, media; fr, frequency. National region: A, Amazon, C, Coast, and H, Highlands; Institution: P, public and Pr, private; Marital status: S, single, M, married, D, divorced, and W, widowed; and Main occupation: St, student and SW, student and worker.

*** *p* < 0.001.

### Measures

The questionnaire had the following sections:

#### Sociodemographic Data

This section included information regarding basic sociodemographic data, such as gender, age, marital status, geographic region, and main occupation.

#### Alcohol Use Disorders Identification Test (Self-Report Version)

It is a 10-item questionnaire that measures dangerous alcohol intake (Babor et al., 2001). Each question is answered indicating the frequency of alcohol consumption and/or the experience of symptoms related to problem consumption on a scale that goes from 0 (“never”) to 4 (“4 or more times a week”), with a maximum score of 40. A high score reflects an increased risk of problem alcohol use, the cut-off point being a score of 8 or more (Conigrave et al., 1995; Berner et al., 2007). The Ecuadorian adaptation of the Spanish version of the Alcohol Use Disorders Identification Test (AUDIT; López et al., 2019) was used, whose Cronbach’s α coefficient for the reliability of internal consistency was 0.816 for men and 0.795 for women.

#### Perceived Stress Scale-14

It measures the degree to which people perceive a lack of control in their daily life, and it has 14 items (Remor, 2006). It is answered according to a 5-point Likert-type scale that ranges from 0 (“never”) to 4 (“very often”). The score ranges from 0 to 56 points. Higher levels of psychological stress represent higher scores. The Ecuadorian adaptation of the Perceived Stress Scale (PSS-14) was used, whose Cronbach’s α coefficient was 0.85 (Ruisoto et al., 2020).

#### Acceptance and Action Questionnaire

The Ecuadorian adaptation of the Acceptance and Action Questionnaire (AAQ-7) was used (Bond et al., 2011; Paladines-Costa et al., 2021). It measures the psychological inflexibility or rigidity in the handling of unpleasant emotions or internal events. It has seven items and is answered on a 7-point Likert-type scale, which ranges from 1 (“never”) to 7 (“always”). Scores vary between 7 and 49. Higher scores represent greater inflexibility or greater tendency to act under the need to control or avoid aversive thoughts, memories, or feelings. Cronbach’s *α* coefficient was 0.919.

### Design and Procedure

A descriptive cross-sectional study was conducted. Data were collected over a period of 3 weeks (November 2015). Participation was confidential and anonymous. At the end, subjects obtained a free summary of individual scores, the purpose of which was to encourage them to answer honestly and increase the response rate. The study was reviewed and approved by the Ethics Committee for Research in Human Beings of the Ministry of Public Health of the Republic of Ecuador in June 2015 (MSP-DIS-2015-0088-O) and was developed following the principles of the Declaration of Helsinki (World Medical Association, 2013). Written informed consent was obtained from all participants.

### Data Analysis

The Statistical Package for the Social Sciences, version 21.0 for Mac (IBM Spain, Madrid, Spain) was used for data analysis. The sample was described through the means and standard deviations (M + SD) for the quantitative variables. For the nominal variables, frequencies and percentages were used. Pearson’s correlation analysis was used to explore the relationship between quantitative variables, and Student’s *t*-test was used to examine the differences between the two independent samples. Effect size was measured by Cohen’s *d* and interpreted following Lakens (2013) and Cohen (1988) guidelines. Shapiro–Wilk test was used to check normality and Levene’s to examine equality of variance across gender groups. In addition, in order to analyze whether there were gender differences in the presence of problematic alcohol consumption as well as whether there were statistically significant differences between the scores for each psychological variable, we used Chi-square test for independence (*χ*^2^) for comparison of frequencies of categorical variables (with Yates’ Continuity Correction and Fisher’s exact test where necessary). Subsequently, an independent hierarchical multiple regression model was conducted to examine the effect of gender (step 1), age (step 2), and transdiagnostic variables: psychological inflexibility or psychological stress (Step 3) on alcohol consumption, controlling for different covariates significantly associated with the outcome. The variance inflation factor (VIF) was used to detect multicollinearity, considering a VIF > 5 as the cut-off point for the collinearity diagnosis. The multiple determination coefficient for multiple regression (*R*^2^) was evaluated together with residual plots to determine the goodness of fit of the data to the regression model and to rule out possible biases. Finally, indirect effect of psychological inflexibility/gender on the effect of psychological stress on alcohol consumption was examined using Process macro version 3.3 (Sheather, 2009) for SPSS (models 4 for conditional mediation analysis and 7 for moderated mediation analysis). The number of bootstrap samples was set at 10,000. Complementary, Baron and Kenny’s (1986) mediational triangle was used to visually display the mediation effects. The general significance level adopted was *p* < 0.05.

## Results

### Gender Differences on Problematic Alcohol Consumption and the Main Transdiagnostic Predictor Variables

Reported alcohol consumption was significantly higher in men (*M =* 7.8; SD *=* 6.15) than women (*M =* 4.81 ± 4.90; *t*_7,903_ = 24.05, *p* < 0.001), showing a medium effect size (Cohen’s *d* = 0.54). Table 2 shows the main gender differences of alcohol use by level of consumption according to AUDIT scores.

**Table 2:** Gender differences on alcohol consumption levels (AUDIT scores).

	Men			Women			*t*	*p*	Cohen’s *d*
	*n* (%)	M	SD	*n* (%)	M	SD			
Non-consumption	530 (32.8)	0	0	1,085 (67.2)	0	0	–	–	–
Prudent	1,417 (42.5)	4.4	1.9	1,914 (57.5)	3.5	1.6	15.24	<0.001	0.52
Risky	1,308 (54.5)	10.9	2.2	1,092 (45.5)	9.8	2.4	11.72	<0.001	0.50
Harmful	243 (71.1)	17.3	1.1	99 (28.9)	17.1	1.1	1.16	0.24	0.18
Dependence	158 (72.8)	24.1	3.8	59 (27.2)	23.2	3.0	1.85	0.07	0.25

AUDIT = 0 = Non-consumption; AUDIT (men) > 0 < 8 = Prudent; AUDIT (women) > 0 < 7 = Prudent; AUDIT (men) ≥ 8 < 15 = Risky; AUDIT (women) ≥ 7 < 15 = Risky; AUDIT ≥ 16 < 20 = Harmful; and AUDIT = 20–40 = Dependence.

A Kruskal-Wallis test revealed a statistically significant difference in the alcohol consumption in the three geographical regions *χ*^2^(2, *N* = 7,905) = 309.07, *p* < 0.001. Alcohol consumption was lower in coast region (*Md* = 3) in comparison with the amazon region (*Md* = 6) and highlands region (*Md* = 6). About gender, Kruskal-Wallis test revealed a statistically significant difference in the male alcohol consumption in the three regions *χ*^2^(2, *N* = 3,656) = 191.44, *p* < 0.001. Alcohol consumption in men was lower in coast region (*Md* = 5) in comparison with the amazon region (*Md* = 8) and highlands region (*Md* = 8); about female alcohol consumption, Kruskal-Wallis test revealed a statistically significant difference in the three regions *χ*^2^(2, *N* = 4,249) = 103.52, *p* < 0.001. Alcohol consumption in women was lower in coast region (*Md* = 3) and amazon region (*Md* = 3) in comparison with highlands region (*Md* = 4).

Finally, gender differences were consistently reported in the two transdiagnostic predictor variables of alcohol consumption. In this regard, women reported significantly higher levels of perceived stress (27.41; SD *=* 6.32) than men (25.34; SD *=* 6.62; *p* < 0.001, Cohen’s *d* = 0.32). Similarly, women reported significantly higher levels of psychological inflexibility (*M =* 23.03; SD *=* 10.35) than men (*M =* 20.76; SD *=* 10.01; *p* < 0.01, Cohen’s *d* = 0.22).

### Effect of Perceived Stress and Psychological Inflexibility on Problematic Alcohol Consumption

The hierarchical multiple regression showed that gender was a significant predictor of alcohol consumption (Step 1; *F*_1,7,903_ = 578.55, *p* < 0.001; *R*^2^ = 0.068). Moreover, gender and age scores (Step 2) were significant predictors (F_2,7,902_ = 300.98, *p* < 0.001; *ΔR*^2^ = 0.003). Finally, gender, age, stress, and psychological inflexibility (F_4,7,900_ = 239.88, *p* < 0.001) scores (Step 3) were also significant predictors (*ΔR*^2^ = 0.038). Residual plots (*x =* standardized predicted value and *y =* standardized regression residual) were randomly dispersed around the horizontal axis, supporting the appropriateness of the regression model (Table 3).

**Table 3:** Hierarchical regressions examining the effect of perceived stress and psychological inflexibility on alcohol consumption.

Regression models (steps and predictors)	*b*	Confidence interval (95%)	*p*	VIF
**Step 1** (*R*^2^ = 0.068)				
Gender	−0.261	−3.235/−2.748	<0.001	1.000
**Step 2** (*R*^2^ = 0.071)				
Gender	−0.257	−3.192/−2.704	<0.001	1.006
Age	0.051	0.046/0.112	<0.001	1.006
**Step 3** (*R*^2^ = 0.108)				
Gender	−0.285	−3.502/−3.017	<0.001	1.031
Age	0.056	0.054/0.119	<0.001	1.007
Perceived stress (PSS-14)	0.080	0.010/0.021	<0.001	1.823
Psychological inflexibility (AAQ-7)	0.133	0.021/0.032	<0.001	1.803

b, unstandardized Beta Coefficient; VIF, Variance inflation factor; PSS-14, Perceived Stress Scale; and AAQ-7, Acceptance and Action Questionnaire.

### Mediation and Moderation Models of Perceived Stress and Psychological Inflexibility on Problematic Alcohol Consumption

**Figure 1:**
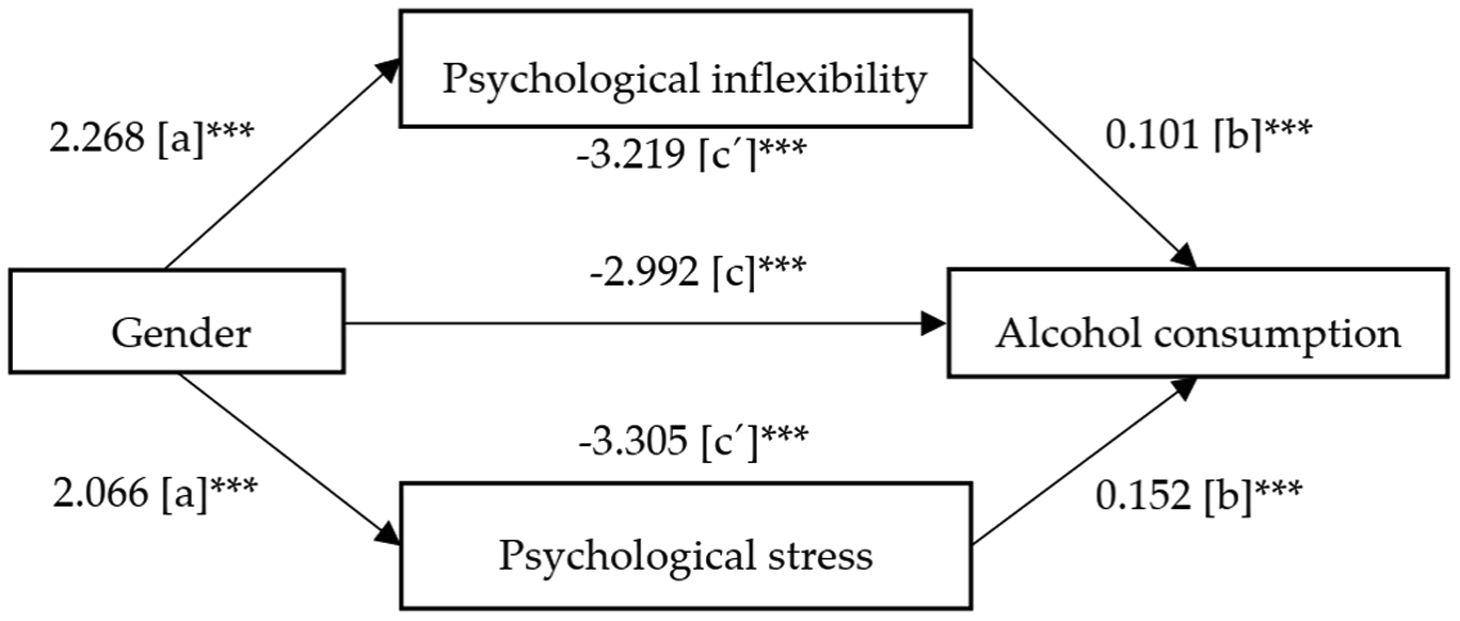
Unstandardized regression coefficients for the mediation effect of (a) psychological inflexibility on the relationship between gender and alcohol consumption and (b) stress on the relationship between gender and alcohol consumption. ^***^p < 0.001.

After controlling for significant covariates in the regression models (gender, age, stress, and psychological inflexibility), following recommendation of Baron and Kenny’s (1986), the Figure 1 visually displays the mediational triangle for the relationship between gender and alcohol consumption mediated by psychological inflexibility and stress.

Gender significantly predicted psychological inflexibility (path a), *b* = 2.268, *t*_(7,903)_ = 9.863, *p* < 0.001, explaining 1.20% of the variance of psychological inflexibility (*p* < 0.001). On the other hand, psychological inflexibility significantly predicted alcohol consumption (path b), *b* = 0.101, *t*_(7,902)_ = 16.820, *p* < 0.001, accounting for 10.04% of the variance (*p* < 0.001). The direct effect of gender on alcohol consumption (path c), ignoring the mediator, was significant, *b* = −2.992, *t*_(7,903)_ = −24.053, *p* < 0.001. Finally, the indirect effect of gender on alcohol consumption (path c’) after controlling for psychological inflexibility as mediator and covariates was significant, *b* = −3.219, *p* < 0.001, 95% confidence interval (CI) ranging from 0.1746 to 0.2836.

On the other hand, gender significantly predicted stress (path a), *b* = 2.066, *t*_(7903)_ = 14.181, *p* < 0.001, explaining 2.48% of the variance of stress (*p* < 0.001). Moreover, stress significantly predicted alcohol consumption (path b), *b* = 0.1518, *t*_(7902)_ = 16.063, *p* < 0.001, accounting for 9.77% of the variance (*p* < 0.001). The indirect effect of gender on alcohol consumption (path c’) after controlling for stress as mediator and covariates was significant, *b* = −3.305, *p* < 0.001, 95% confidence interval (CI) ranging from 0.2556 to 0.3742.

**Figure 2:**
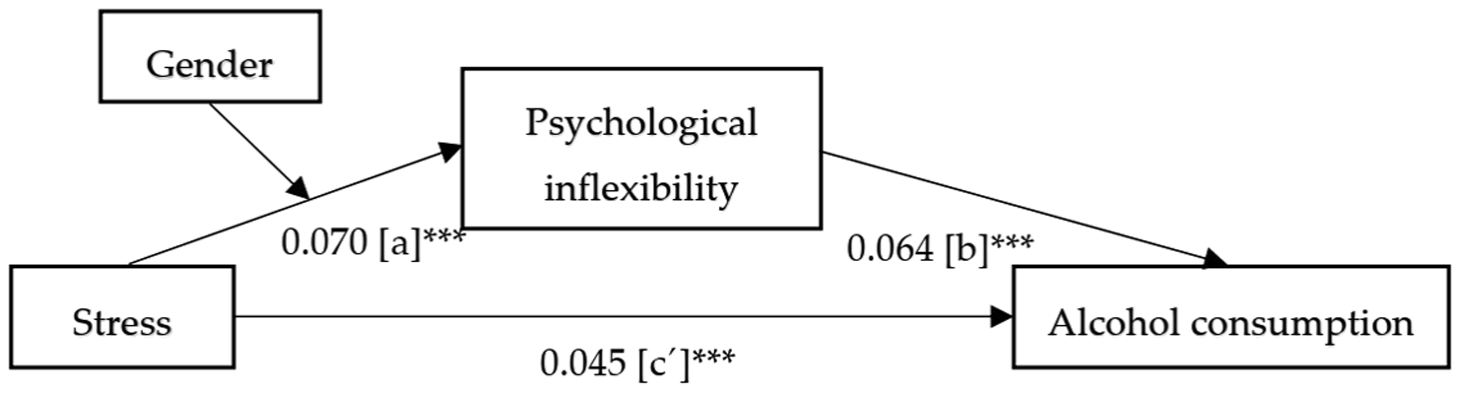
Unstandardized regression coefficients for the mediation effect of psychological inflexibility on the relationship between stress and alcohol consumption, moderated by gender. ^***^p < 0.001. Moderated mediation index = 0.004 CI [0.001–0.008].

In a next step, and given that psychological inflexibility and stress are variables that mediate the effect of gender on alcohol consumption (as has just been indicated), we analyzed the mediation effect of psychological inflexibility on the relationship between stress and alcohol use, as well as the moderating effect of gender (Figure 2).

First, stress significantly predicted psychological inflexibility, *b* = 0.944, *t*_(7901)_ = −2.842, *p* < 0.001, 95% CI [0.861–1.027], and gender predicted psychological inflexibility, *b* = −1.735, *t*_(7901)_ = −2.410, *p* < 0.05, 95% CI [−3.146 to −0.324]. Moreover, the interaction between stress and gender predicted psychological inflexibility (path a), *b* = 0.070, *t*_(7901)_ = 2.626, *p* = 0.010, 95% CI [0.018–0.122], explaining 44.96% of the variance of psychological inflexibility (*p* < 0.001). On the other hand, psychological inflexibility significantly predicted alcohol consumption (path b), *b* = 0.064, *t*_(7902)_ = 7.703, *p* < 0.001, 95% CI [0.048–0.081]. Direct effect of stress on alcohol consumption (path c´), ignoring the mediator and moderator, was also significant, *b* = 0.045, *p* < 0.001, 95% CI ranging from 0.019 to 0.070, explaining 2.38% of the variance of alcohol consumption (*p* < 0.001). Finally, the indirect conditional effect of stress (path c) after controlling for psychological inflexibility as mediator and gender as moderator was significant for men (*b* = 0.065, 95% CI [0.048–0.083]) and women (*b* = 0.070, 95% CI [0.051–0.089]). The relationship between the indirect effect and the moderating variable, gender, was statistically significant (moderated mediation index = 0.004, 95% CI, 0.001–0.008).

## Discussion

The aim of this study was to examine gender differences in psychological stress and psychological inflexibility as transdiagnostic predictors of problematic alcohol consumption in a large sample of college students in Ecuador. To our knowledge, this is the first and largest study carried out in Ecuador examining alcohol consumption from both a transdiagnostic and gender perspective. Results have shown higher rates of alcohol consumption among men than women (of every 10 students with harmful alcohol consumption or dependence, seven are men), and higher levels of stress and psychological inflexibility among women than men. Interestingly, psychological inflexibility and stress mediated the effect of gender on alcohol consumption. Moreover, the mediation of psychological inflexibility between stress and alcohol consumption is positive and strong for both men and women, that is, gender moderated this mediation.

Firstly, men reported higher rates of alcohol consumption than women, being such result consistent with previous studies (UNODC, 2017; Gavurova et al., 2020; Villarosa-Hurlocker et al., 2020). Traditionally, research highlights that men drink more per drinking episode, that is, more often and over longer periods of time than women, while the last frequently use protective behavioral strategies (LaBrie et al., 2011). In this vein, Nolen-Hoeksema and Hilt (2006) found that men, compared to women, are more likely to be exposed to some risk factors, such as fewer social penalties for drinking and more positive expectations regarding alcohol consumption, among others as well as they might perceive alcohol use as a more acceptable activity because such consumption could be a part of their gender role (Perrotte et al., 2020). By contrast, the manifestation of alterations in cognitive and motor functioning, even with low doses of alcohol, in addition to its negative consequences on reproductive health, can dissuade most women from alcohol consumption, which would explain the presence of lower rates of alcohol use disorders and related problems, compared to men (Nolen-Hoeksema, 2004). Consumption in women, although lower than in men, according to Norberg et al. (2010), might be explained by social anxiety levels, a risk factor for them, who are more likely to report drinking in negative situations to deal with aversive emotions. In this regard, Engs and Hanson (1990) found that a higher percentage of college men are more likely than women to drink, to drink more often, to consume more, and to experience more drinking problems.

As has previously reported, several transdiagnostic factors have traditionally influenced severity of AUD, such as stress and psychological inflexibility. In particular, stress appears to be a serious problem for college students, who represent a very vulnerable group of the population (Robotham and Julian, 2006; Saleh et al., 2017) that commonly meet several health complaints and symptoms of depression, mood swings, and anxiety (Barbosa-Leiker et al., 2012). In the present study, women reported significantly higher levels of perceived stress than men students did. These findings are consistent with the results obtained in precedent research conducted among college students (Tavolacci et al., 2013; Varghese et al., 2015; Cavallo et al., 2016; Olfert et al., 2019; Seedhom et al., 2019; Choi, 2020; Meckamalil et al., 2020), and such gender differences may be explained due to women usually choose emotional-based coping styles (Tamres et al., 2002) as well as due to the presence of reduced stress resilience and coping resources (Kizhakkeveettil et al., 2017), leading to lower levels of wellbeing (Innes, 2017). Taking into account that there are several factors involved, more research is needed to understand and determine the differences in stress between the genders.

On the other hand, Psychological inflexibility is the avoidance of painful internal experiences, such as memories, thoughts, emotions, sensations, and representing a difficulty to act consistently according to significant personal values (Serowik and Orsillo, 2019). There is more and more research that supports the idea that psychological inflexibility is a transdiagnosis variable present in various psychological disorders (Kashdan and Rottenberg, 2010; Bond et al., 2011; Levin et al., 2014). For our purpose, psychological inflexibility has been theorized to play a role in the cause and maintenance of substance use disorders (Levin et al., 2012, 2014). In the present study, women reported significantly higher levels of psychological inflexibility than men students did, being such results consistent with precedent research (Levin et al., 2012; Mendoza et al., 2016; Serrano-Ibáñez et al., 2021). This could be explained due to the differences between men and women regarding their emotional processing (Whittle et al., 2011), as women tend to experience more negative affect than men and report more frequent and intense negative emotions (Brebner, 2003), which can lead them to avoid these emotions. It could also be explained due to the fact that higher levels of psychological inflexibility in women than in men may be related to traditional gender roles established in the Latino cultural context (Mendoza et al., 2016).

Furthermore, hierarchical regressions determined than psychological stress and inflexibility were significant predictors of alcohol consumption. Precedent studies (Kingston et al., 2010; Levin et al., 2012) also highlight the importance of psychological inflexibility in alcohol consumption. Levin et al. (2014) found that this variable was significantly related to having a comorbid depressive, anxiety, or substance use (including alcohol) disorder. In contrast, Serowik and Orsillo (2019) found little evidence for a unique association between inflexibility in alcohol consumption. Regarding psychological stress, it has been linked to an increased alcohol consumption (Sebena et al., 2012; Collins et al., 2014; Böke et al., 2019) in college students, and therefore, it is possible that students who are stressed drink alcohol as an experiential avoidance strategy. In view of the importance of knowing the variables that explain alcohol consumption and the variety of results found in the aforementioned studies, more research is needed.

The mediation model of psychological inflexibility and stress on the effect of gender on alcohol consumption was statistically significant. These results suggest for the first time in the literature that higher levels of psychological inflexibility and stress may play a role in alcohol consumption among college students, regardless of gender. Students (men and women) who consume higher amounts of alcohol do so not only because they experience negative emotional states, but also because they experience higher levels of stress. Eisenbeck et al. (2019) found that psychological inflexibility was related to psychological stress. Apparently, college students consume alcohol in order to avoid unpleasant thoughts, emotions, and sensations (Zgierska et al., 2009); in other words, those who drink motivated to change their unpleasant internal states to reduce their negative affects, are at greater risk of high alcohol consumption (Reynolds et al., 2015). Alcohol consumption, when it serves inflexible tendencies, could increase the level of unwanted thoughts and feelings, being possible that high levels of psychological inflexibility generate an endless cycle guided by unpleasant experiences and their avoidance, which in turn generates more unpleasant experiences. If a person believes that feeling stress or anxiety is a negative thing, they are likely to use problematic coping strategies, such as avoidance, substance abuse, or other equally harmful ones. (Leahy, 2018). To date, there are no studies that have analyzed these same relationships, although there exist precedent research that includes other interesting transdiagnostic variables. For example, Levin et al. (2012) found that psychological inflexibility mediated the relationship of psychological distress to alcohol-related problems, and Pedrelli et al. (2011) found a moderated relationship between depressive symptoms and daily alcohol consumption. Moreover, Devynck et al. (2019) found a strong positive association between repetitive negative thinking and alcohol use or alcohol-related problems. Negative urgency (propensity to engage in impulsive behavior while experiencing distress) also explains the association between anxiety symptomatology and both alcohol and cannabis use problems (Wolitzky-Taylor et al., 2016). Nonetheless, more studies are required to reach conclusive results in this field.

The effect of stress on alcohol consumption after controlling for psychological inflexibility as mediator and gender as moderator was significant for both men and women students. In other words, the mediation of psychological inflexibility between stress and alcohol consumption is positive and strong for both men and women, even higher in women. It could be explained because women reported significantly higher levels of both psychological inflexibility and stress than men students did. Likewise, Kheirabadi et al. (2021) and Barenz (2017) revealed that psychological inflexibility positively mediates the relationship between stress and substance abuse. However, to our knowledge, there are no investigations that have studied the relationships between these variables. Therefore, our study adds to the previous literature the influence of gender in the relationship between stress and psychological inflexibility, as transdiagnostic variables, in the consumption of alcohol among college students.

This transdiagnostic approach opens up new ways of assessing and classifying AUD, suggesting alternatives in the understanding of the processes involved, and provides a new theoretical foundation on the initiation and maintenance, as well as the clinical treatment and recovery of these disorders (Dalgleish et al., 2020). Overall, this novel emotional vulnerability model includes a set of key clinical factors underlying alcohol use and different emotional conditions (Leventhal and Zvolensky, 2015). However, to date, no research had yet examined the relationship between both transdiagnostic constructs (psychological stress and psychological inflexibility) and problematic alcohol consumption. There is still much to investigate henceforth to generate true innovations and avoid conceptual biases to deliver a transdiagnostic paradigm shift that can improve assessment protocols and clinical care (Fusar-Poli et al., 2019).

Several limitations of this study merit mention. Firstly, statistically significant differences might be sensible to the large size of the sample. In addition, results might be confound by university culture, since differences in alcohol consumption were reported between the three main Ecuadorian regions in men and women, as expected based on previous studies (CONSEP, 2014). Moreover, results of this study must be considered with caution since cross-sectional design limits the capacity to stablish causal relationships. Future studies would benefit from longitudinal design to further explore changes on psychological inflexibility, stress, and alcohol consumption over time. Finally, self-reported data may be subject to desirability.

## Conclusion

Results of this study support psychological stress and psychological inflexibility as critical transdiagnostic variables related to increased rates of alcohol consumption among Ecuadorian college students. Furthermore, psychological inflexibility mediates the negative impact of psychological stress on alcohol consumption, with a higher degree among women. These findings contribute to the development of transdiagnostic comprehensive programs, which encompasses promotive, preventive, and treatment services, to alleviate the burden of alcohol, including gender differences, and to enrich the growing research on alcohol consumption in this population.

## Data Availability Statement

The raw data supporting the conclusions of this article will be made available by the authors, without undue reservation.

## Ethics Statement

The studies involving human participants were reviewed and approved by MSP-DIS-2015-0088-O. The patients/participants provided their written informed consent to participate in this study.

## Author Contributions

PR and SV-G: conceptualization and funding acquisition. PR, CL-N, and RS-P: methodology and data curation, RS-P: writing—original draft preparation. PR and CL-N: writing—review and editing. All authors have read and agreed to the published version of the manuscript.

## Funding

This study was funded by the Universidad Técnica Particular de Loja (Ecuador) and the National Council for the Control of Narcotic Drugs and Psychotropic Substances (CONSEP), grant number PROY.PSC.1055. Additional funding was provided by the European Union—Next Generation EU through the Grant for the Requalification of the Spanish University System for 2021–2023 at the Public University of Navarra (Resolution 1402/2021). The funders had no role in the study design, data collection and analysis, decision to publish, or preparation of the manuscript.

## Conflict of Interest

The authors declare that the research was conducted in the absence of any commercial or financial relationships that could be construed as a potential conflict of interest.

## Publisher’s Note

All claims expressed in this article are solely those of the authors and do not necessarily represent those of their affiliated organizations, or those of the publisher, the editors and the reviewers. Any product that may be evaluated in this article, or claim that may be made by its manufacturer, is not guaranteed or endorsed by the publisher.
